# Genetic and Lifestyle-Related Factors Influencing Serum Hyper-Propionylcarnitine Concentrations and Their Association with Metabolic Syndrome and Cardiovascular Disease Risk

**DOI:** 10.3390/ijms242115810

**Published:** 2023-10-31

**Authors:** Yong-Hwa Lee, Sunmin Park

**Affiliations:** 1 Department of Cosmetic Biotechnology, Hoseo University, Asan 31499, Republic of Korea; yhleef@hoseo.edu; 2 Department of Food and Nutrition, Institute of Basic Science, Obesity/Diabetes Research Center, Hoseo University, Asan 31499, Republic of Korea

**Keywords:** serum propionylcarnitine concentrations, serum branched-chain amino acid concentration, dietary branched-chain amino acid, polygenic risk score, dietary pattern

## Abstract

The genetic and environmental determinants of serum propionylcarnitine concentrations (PC) remain largely unexplored. This study investigated the impact of genetic and environmental factors on serum propionylcarnitine levels in middle-aged and elderly participants from the Ansan/Ansung cohort of the Korean Genome and Epidemiology Study. Our goal was to understand the role of PC on the risk of metabolic syndrome (MetS) leading to cardiovascular disease, particularly concerning branched-chain amino acid (BCAA) metabolism. We analyzed participants’ demographic, lifestyle, and biochemical data with and without MetS. Serum metabolite concentrations, including carnitine, acylcarnitine, and amino acid concentrations, were measured, and the components of MetS were evaluated. Genetic variants associated with low and high PC were selected using genome-wide association studies after adjusting for MetS-related parameters. Further, genetic variants and lifestyle factors that interacted with the polygenic risk score (PRS) were analyzed. Participants with MetS were older and less educated, and their alcohol intake was higher than non-MetS participants. PC was significantly associated with the MetS risk and increased the serum levels of BCAAs and other amino acids. Higher PC positively correlated with MetS components, insulin resistance, and cardiovascular risk factors. Intake of calcium, sodium, and vitamin D were inversely associated with PC, but coffee consumption was positively linked to PC. Multiple C2 And Transmembrane Domain Containing-1 (*MCTP1*)_rs4290997, Kinesin Family Member-7 (*KIF7*)_rs2350480, Coagulation Factor-II (*F2*)_rs2070850, Peroxisomal Biogenesis Factor-3 (*PEX3*)_rs223231, TBC1 Domain Family Member-22A (*TBC1D22A*)_rs910543, and Phospholipase A2 Group-IV-C (*PLA2G4C*)_rs7252136 interact with each other to have a threefold influence on PC. The PRS for the six-genetic variant model also interacted with age; the diet rich in beans, potato, and kimchi; and smoking status, influencing PC. In conclusion, elevated PC was associated with MetS and cardiovascular disease risk, suggesting their potential as disease biomarkers.

## 1. Introduction

Metabolic syndrome (MetS) comprises a group of metabolic irregularities, such as high blood pressure, central obesity, hyperglycemia, and dyslipidemia, frequently manifesting concurrently. This co-occurrence elevates the likelihood of developing cardiovascular disease, stroke, and type 2 diabetes [1]. MetS is a prevalent and concerning health condition with a significant global impact, and its incidence has been on the rise over recent decades [1]. Although the precise etiology of MetS remains incompletely understood, it is widely recognized that a combination of genetic, lifestyle, and environmental factors contributes to its development [2,3]. 

To understand the intricate relationship between MetS and serum propionylcarnitine, it is important to delve into the roles of these key components. Serum propionylcarnitine is a metabolite derived from the conversion of propionic acid by carnitine acyltransferase, and it holds a critical position in mitochondrial energy production. This metabolic process is a vital part of the energy production machinery within cells, involving the transport of various acyl groups across the mitochondrial membrane to fuel the generation of cellular energy [4]. Notably, propionylcarnitine production is tightly regulated under normal circumstances, but metabolic abnormalities, such as reduced beta oxidation of fatty acids in the mitochondria, or a deficiency in propionyl-CoA carboxylase, can lead to its accumulation in the bloodstream [4,5]. 

An intriguing aspect of propionylcarnitine is its connection to branched-chain amino acids (BCAAs). BCAAs, which include leucine, isoleucine, and valine, are essential amino acids that serve as fundamental building blocks for protein synthesis and play pivotal roles in energy production [6]. Leucine, in particular, is a potent activator of the mammalian target of the rapamycin (mTOR) signaling pathway, a central regulator of protein synthesis. Additionally, it enhances insulin sensitivity in muscle cells, further promoting protein synthesis [7]. Isoleucine and valine contribute to glucose uptake in skeletal muscle cells and participate in energy metabolism, indirectly supporting protein synthesis and preventing muscle protein breakdown [8]. Recent research studies have pointed to a potential connection between elevated serum BCAA levels and an increased risk of MetS and cardiovascular disease [6,9]. This association is complex and likely influenced by various factors, including insulin resistance, disrupted energy metabolism, and inflammation [10]. 

Serum metabolite concentrations can be influenced by both genetic and environmental factors [11,12]. Genetic variations can affect the enzymes and pathways involved in the metabolism of propionylcarnitine, potentially leading to variations in its concentration in the blood [11]. The genetic impact is represented with polygenic risk score (PRS) to understand its complex relationship with MetS risk. However, to date, there is no study on the genetic association of serum propionylcarnitine concentrations influencing MetS. Considering this complex interplay between serum propionylcarnitine concentration and BCAA metabolism, this study aims to investigate the impact of genetic and environmental factors on serum propionylcarnitine levels. We hypothesize that serum propionylcarnitine concentrations, influenced by genetic and environmental factors, play a pivotal role in the development of metabolic syndrome (MetS). Furthermore, the presence of PRS affecting propionylcarnitine metabolism contributes to variations in MetS risk. The hypothesis was examined in middle-aged and elderly participants from the Ansan/Ansung cohort of the Korean Genome and Epidemiology Study (KoGES). By investigating this hypothesis, we can gain valuable insights into the mechanisms underlying MetS and the potential avenues for prevention and intervention.

## 2. Results

### 2.1. Characteristics of Participants with and without MetS

Participants with MetS were older than those without MetS, and age was positively associated with the presence of MetS. The gender distribution between the MetS and non-MetS groups was not significantly different (Table 1). Participants with MetS were less educated than those without MetS. The distribution of participants residing in urban and rural areas was similar between the MetS and non-MetS groups (Table 1). With respect to lifestyles, participants with MetS had a slightly higher alcohol intake (11.2 g/day) compared to those without MetS (9.41 g/day), and a positive association was exhibited between alcohol consumption and MetS (Table 1). However, the percentage of participants with various levels of physical activity was similar in the MetS and non-MetS groups, and the distribution of nonsmokers, former smokers, and current smokers was not significantly different between the two groups (Table 1). 

### 2.2. Serum Carnitine, Acylcarnitine, and Amino Acid Concentrations

There were no significant differences in the serum carnitine levels between the MetS and non-MetS groups (Table 2). On the other hand, serum acetylcarnitine levels were significantly lower in the MetS group (odds ratio (OR): 0.774, 95% CI: 0.612–0.979) and serum propionylcarnitine levels were significantly higher (OR: 1.454, 95% CI: 1.163–1.817), as were the levels of valine, isoleucine, leucine, alanine, and tyrosine (OR ranging from 1.498 to 1.983) (Table 2). Although the serum methionine concentrations were higher in the MetS than in the non-MetS group, there was no significant association of methionine with MetS risk. Threonine, lysine, and tryptophan showed no significant differences between the two groups (Table 2). Dietary intake of BCAA, including valine, isoleucine, and leucine contents, was lower in the MetS than in the non-MetS groups (*p* < 0.001). However, there was no significant association between dietary BCAA and MetS risk (Table 2). Dietary methionine and tyrosine contents did not differ between the MetS and non-MetS groups (Table 2).

### 2.3. Incidence of MetS and Its Components According to Serum Propionylcarnitine Concentrations

**Figure 1:**
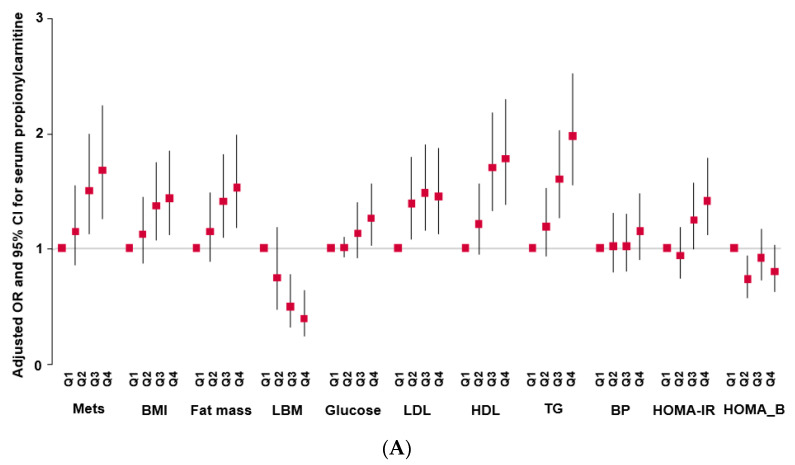

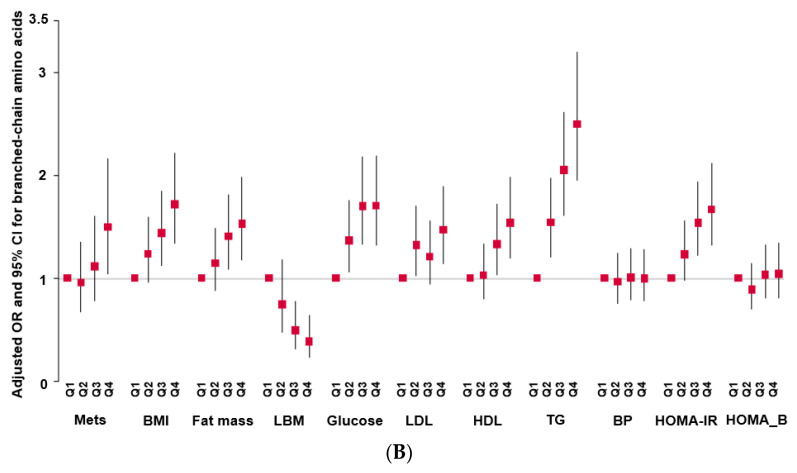
Adjusted odds ratio (OR) and 95% confidence intervals (CIs). (**A**): Serum propionylcarnitine concentrations with the quintiles of metabolic syndrome (MetS) and its components. (**B**): serum branched-chain amino acid concentrations with the quintiles of MetS and its components.

MetS incidence was higher in the high-propionlycarnitine (High-PC) than low-propionlycarnitine (Low-PC) groups, at *p* < 0.01, and there was a statistically significant association of MetS risk with serum propionlycarnitine levels (ORs = 1.389; 95% CI = 1.107–1.742; Table 3, Figure 1A). Among the MetS components, BMI, waist circumference, and body fat were higher in the High-PC than in the Low-PC group, and propionlycarnitine was also positively associated with BMI, waist circumference, and body fat (Table 3; Figure 1A). Conversely, LBM was lower in the High-PC than in the Low-PC group, and it was inversely associated with serum propionylcarnitine concentrations (Table 3; Figure 1A). Serum propionylcarnitine concentrations were not significantly linked to serum glucose and insulin concentrations at 0 and 60 min after oral 75 g glucose loading (Table 3, Figure 1A). However, serum glucose and insulin concentrations at 120 min were higher in the High-PC than in the Low-PC group, and they were positively associated with serum propionlycarnitine concentrations (Table 3). Higher serum propionylcarnitine concentrations raised the risk of insulin resistance 1.345-fold. However, the serum propionylcarnitine concentrations did not affect beta cell function/insulin secretion (95% CI = 1.112–1.626). Serum HDL-C concentrations were inversely associated with serum propionylcarnitine concentrations, and serum triglyceride concentrations were positively associated with serum propionylcarnitine concentrations (Table 3, Figure 1A). SBP and DBP were also higher in the High-PC than in the Low-PC group, and they were positively associated with serum propionylcarnitine concentrations, by 1.3 and 1.9 times, respectively (Table 3). Consistent with the association of serum propionylcarnitine concentrations in exacerbating the risk of MetS and its components, the incidence of cardiovascular disease was higher in the High-PC than in the Low-PC group. Cardiovascular disease was positively linked, 1.9-fold, to serum propionylcarnitine concentrations (Table 3, Figure 1A). These findings revealed that increased serum propionylcarnitine concentrations were a potential risk factor for MetS and cardiovascular disease.

Serum BCAA concentrations were also associated with MetS, BMI, fat mass, serum glucose, low-density lipoprotein cholesterol (LDL-C), hypo-HDL, TG concentrations, and HOMA-IR in the quartile category (Figure 1B). Among the parameters, the serum TG concentration showed a significant increase in the high-PRS group (Figure 1B). 

### 2.4. Serum Propionylcarnitine Concentrations According to Nutrient Intake

The intake of energy and macronutrients (carbohydrates, fat, and protein) did not differ between the Low-PC and High-PC groups, and there was no significant association between energy and macronutrients and serum propionylcarnitine concentrations (Table S1). The intake of BCAA and its components did not differ between the Low-PC and High-PC groups (Table S1). Fiber and vitamin C intake did not differ between the Low-PC and High-PC groups. Calcium and vitamin D intake was lower and sodium intake was higher in the High-PC group compared to the Low-PC group. However, no significant association existed between these nutrients and serum propionylcarnitine concentrations (Table S1). Coffee intake was higher in the High-PC group than in the Low-PC group, and it had a positive association with serum propionylcarnitine concentrations (Table S1). Caffeine intake was also higher in the High-PC group than in the Low-PC group, but no significant association existed between caffeine intake and serum propionylcarnitine concentrations (Table S1). Food intake measured by SQFFQ was clustered into four dietary types by principal component analysis (PCA), namely, a Korean balanced diet (KBD); a diet high in noodles, bread, and fast food (NBFD); a diet rich in beans, potato, and kimchi (BPKD); and a rice-main diet (RMD). No variation was seen between the low-PC and high-PC groups based on the Korean balanced diet, NBFD diet, and rice-main diet (Table S1). 

### 2.5. Genetic Variants Associated with Serum Propionylcarnitine Concentrations

The statistical significance levels of the genetic variants associated with serum propionylcarnitine concentrations were presented using a Manhattan plot (Supplementary Figure S1A). The genome inflation of the selected genetic variants was determined using the lambda value by comparing the observed and expected *p*-values. The lambda was 1.016, indicating no inflation of the genetic variants for serum propionylcarnitine concentrations (Supplementary Figure S1B). 

The 10 genetic variants linked to serum propionylcarnitine concentrations were selected from the GWAS at the significance level 5 × 10^−5^. The selected genetic variants had minor allele frequency (MAF) > 0.01 and an HWE *p*-value > 0.295, indicating that the selected genetic variants passed the SNP quality threshold (Table 4). *CELSR2_*rs629301 was located in 3’-UTR, and *H-Cadherin (CDH13)_*rs9933938, *Phospholipase A2 Group IV C (PLA2G4C)_*rs7252136, and *TBC1 domain family member 22A (TBC1D22A)_*rs910543 were presented within or affected a transcript (mRNA). The other six genetic variants were located in the intron area (Table 4). Three genetic variants (*Follistatin-like 4 (FSTL4)_*rs153197, *PLA2G4C_*rs7252136, and *TBC1D22A*_rs910543) were negatively associated with serum propionylcarnitine concentrations, and seven genetic variants were positively linked to it (Table 4). Among the 10 genetic variants, *Chemerin-like receptor 1 (CMKLR1)_rs7315943* was noteworthy due to its high OR of 3.102, indicating a strong association with serum propionylcarnitine concentration (Table 4).

### 2.6. Binding Free Energy of Food Components to Wild and Mutated Types of CELSR2_rs629301

The wild (T) and mutated (G) types of *CELSR2*_rs629301 had different binding free energy to the 20,000 food components evaluated, and Table S2 presents the food components with low binding free energy with the wild and mutated types of *CELSR2*_rs629301. Flavonoids and anthocyanins, including apigenin, procyanidin, plantacyanin, quercetin, kaempferol, ellagic acid, and gallic acid, and their glycones lowered binding energy in both wild and mutated types (Table S2). The wild (T) and mutated (G) types of *CELSR2*_rs629301 (−12.0~−8.3) showed similar energy to some food components (−12.0~−8.3). Some food components had lower binding energy in wild or mutated types (Table S2). The results suggested that the food components selected to lower binding energy to the *CELSR2*_rs629301 site could modulate the serum hyper-propionylcarnitine concentration risk. Goyaglycoside g was found in *Mormodica charantia* extract and reported to reduce insulin resistance. Goyaglycoside g had lower binding energy with the wild (T) type than the mutated (G) type (Table S2, Figure S2). Goyaglycoside g had more conventional hydrogen bonds and interpolated charge with the wild (T) type than with the mutated type of *CELSR2*_rs629301. The effect of Goyaglycoside g on serum hyper-propionylcarnitine concentration risk might be better in the wild type of *CELSR2*_rs629301 than the mutated one (Figure S2).

### 2.7. PRS of Genetic Variants Selected with Their Interaction to Influence Serum Hyper-Propionylcarnitine Concentrations

**Figure 2:**
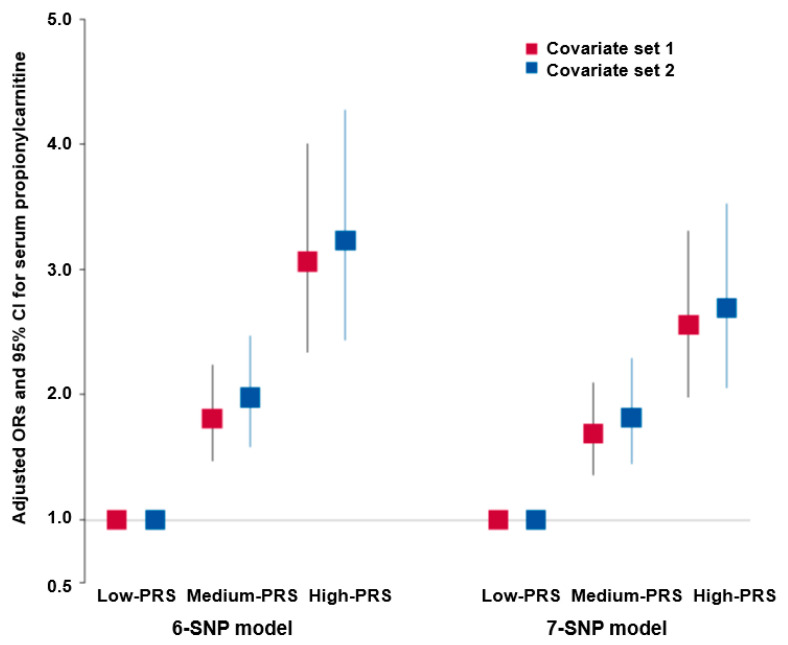
Adjusted odds ratio (ORs) and 95% confidence intervals (CIs) of serum hyper-propionylcarnitine concentration risk with the polygenic risk scores (PRSs) of 6- and 7-single-nucleotide polymorphism (SNP) models with SNP–SNP interactions. The optimal models for generalized multifactor dimensionality reduction analysis were determined for both 6-SNPs and 7-SNPs by adding the risk alleles of the respective 6- and 7-SNPs. Subsequently, calculated polygenic risk scores (PRSs) were categorized into 3 groups for each model: low—PRS (0–5 for 6-SNP and 0–6 for 7-SNP), medium—PRS (6–7 for 6-SNP and 7–8 for 7-SNP), and high—PRS (≥8 for 6-SNP and ≥9 for 7-SNP). Logistic regression analysis was conducted to calculate adjusted odds ratios (ORs), taking into account various covariates, including age, gender, residence areas, income, education, energy intake, smoking status, physical activity, alcohol intake, and the survey year. In the logistic regression, the reference group used consisted of individuals with low PRS. The figures depict the adjusted ORs for the 6- and 7-SNPs using red and blue boxes, respectively, with lines crossing these boxes indicating the corresponding 95% confidence intervals (CIs).

Table S3 presents the interactions between the genetic variants significantly affecting serum propionylcarnitine concentrations. Genetic variants for the PRS in the 6-SNP model met the selection criteria. The genetic variants in the 6-SNP model included *Multiple C2 and Transmembrane Domain Containing-1 (MCTP1)*_rs4290997, *Kinesin Family Member-7 (KIF7)*_rs2350480, *F2*_rs2070850, *Peroxisomal Biogenesis Factor-3 (PEX3)*_rs223231, *TBC1D22A*_rs910543, and *PLA2G4C*_rs7252136. The 7-SNP model included the variant *FSTL4*_rs153197 in addition to those in model 6 (Table S3). The PRSs of the genetic variants in models 6 and 7 were positively associated with serum propionylcarnitine concentrations. Although *CMKLR1_rs7315943* showed the highest ORs, it did not interact with other genetic variants to affect serum propionylcarnitine concentrations (Table S3). The PRS of the 6-SNP and 7-SNP models were positively associated (3.229- and 2.693-fold, respectively) with serum propionylcarnitine concentrations after adjusting with the covariate set 2 (Figure 2). The results suggested that the genetic variants in the 6-SNP model had better interaction than those in the 7-SNP model. The PRS of the 6-SNP model was therefore selected for the genetic interaction with lifestyle factors. 

**Figure 3:**
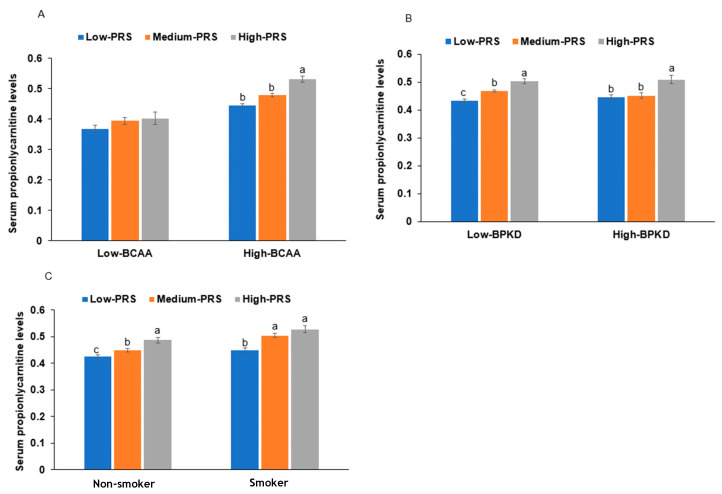
The proportion of individuals with serum hyper-propionylcarnitine concentrations with the polygenic risk scores (PRSs) of the 6-single-nucleotide polymorphism (SNP) model. (**A**): Serum branched-chain amino acid concentration (cutoff: 500 μM). (**B**): Diets high in beans, potatoes, and kimchi (BPKD, cutoff: 33rd percentile). (**C**): Smoking status: low PRS (0–5), medium PRS (6–7), and high PRS (≥8) in the 6-SNP model. a,b,c: different letters on the bars indicate significant differences among the PRS groups in Tukey’s test at *p* < 0.05.

Interestingly, the serum BCAA concentrations had an interaction with serum propionylcarnitine concentrations influencing the PRS. At high serum BCAA concentrations, serum propionylcarnitine concentrations were much higher in the high-PRS than in the low-PRS group (Figure 3A). However, at low serum BCAA concentrations, there was no significant effect of the PRS on serum propionylcarnitine concentrations (Figure 3A). 

### 2.8. PRS Interaction with Lifestyle Factors

Among the four dietary patterns, the BPKD, rich in beans, potato, and kimchi, interacted with the PRS to influence serum propionylcarnitine concentrations (Table 5). The participants consuming a high BPKD had a lower PRS impact, especially in the medium-PRS group, but BPKD cannot overcome the high-PRS impact on serum propionylcarnitine concentrations (Figure 3B). However, other diet types did not have any interactions with PRS. The participants in the high-BPKD group had a lower BCAA intake (1.05 ± 0.11 g/day for low BPKD and 1.02 ± 0.06 g/day for high BPKD; *p* = 0.04). BCAAs may affect serum propionylcarnitine concentrations indirectly. Smoking also interacted with the PRS, and in participants who were smokers, the serum propionylcarnitine concentrations were much higher in each PRS group than in nonsmokers (Figure 3C). This suggested that smoking interacted with the PRS to exacerbate serum propionylcarnitine concentrations.

## 3. Discussion

The results of this study elucidate the potential significance of serum propionylcarnitine concentrations as a marker for MetS and highlight the intricate interplay between genetic and environmental factors in its regulation. Our findings revealed that individuals with MetS exhibited higher serum propionylcarnitine levels. This elevation in propionylcarnitine concentration was also accompanied by increased levels of BCAAs, alanine, tyrosine, and methionine. Furthermore, we observed a positive association between serum propionylcarnitine levels and MetS components, including insulin resistance, elevated glucose levels, and unfavorable lipid profiles. This association, particularly with insulin resistance, aligns with previous research on the role of carnitine metabolism in glucose homeostasis.

Propionic acid, a precursor of propionylcarnitine, is released from dietary sources, gut microbiota metabolites, and BCAA metabolites [13]. Propionic acid is converted to propionyl coenzyme (CoA), which is metabolized into succinyl-CoA and enters the tricarboxylic acid (TCA) cycle. Propionyl CoA can also be transformed into propionylcarnitine, participating in the beta oxidation of fatty acids for energy production, primarily in the liver and kidneys [14,15]. However, despite its role in enhancing energy production, elevated serum propionylcarnitine concentrations have been reported to indicate metabolic disorders, such as propionic acidemia and obesity [14,15]. This is consistent with the findings of our study, wherein the results demonstrated a positive association between serum propionylcarnitine levels and components of MetS, including BMI, waist circumference, body fat, dyslipidemia, and hypertension. This suggests that increased serum propionylcarnitine levels may indicate disruptions in mitochondrial function and fatty acid metabolism, contributing to insulin resistance and the development of MetS. It is important to note that propionylcarnitine may act as an antioxidant in the liver and heart in spontaneously hypertensive rats [16]. Furthermore, its intake has been shown to reduce body weight and hyperinsulinemia in obese Zucker rats [17] and decrease cardiovascular risk by improving mitochondrial function in rats [18]. These results, in addition to our results, appear to suggest that increases in serum propionylcarnitine concentrations could indicate decreases in tissue propionylcarnitine contents, potentially leading to disruptions in mitochondrial function and fatty acid metabolism and contributing to insulin resistance and the development of MetS.

The relationship between serum propionylcarnitine concentrations and BCAAs has yet to be fully understood. However, both are associated with metabolic disorders. Several potential mechanisms have been suggested. BCAAs in the cell can be metabolized in skeletal muscle to produce branched-chain keto acids (BCKAs) via transaminase [6]. These BCKAs can further be converted to propionyl-CoA, along with other intermediates, which are then converted to propionylcarnitine for mitochondrial transport and energy production [19]. However, when both BCAAs and propionylcarnitine enter the bloodstream, it could lead to mitochondrial function overload, which may be associated with metabolic disorders. Our study demonstrated that serum BCAAs and propionylcarnitine concentrations were positively associated with MetS risk, increasing it 1.45- and 1.53-fold, respectively. Further research is needed to completely comprehend the association between serum propionylcarnitine and BCAA concentrations, but existing evidence suggests a link mediated by impaired BCAA metabolism and mitochondrial dysfunction.

The connection between BCAA intake, serum BCAA levels, and MetS is a complex and evolving area of research. Serum BCAA concentrations have been shown to be positively associated with insulin resistance, cardiovascular diseases, obesity, and type 2 diabetes in most studies [20], and this is consistent with the present study. However, the relationship between BCAA intake and insulin resistance remains controversial [21]. Some studies suggest that BCAA intake is essential for muscle growth and repair and can improve insulin sensitivity [22]. However, other studies have shown that BCAA intake can promote the production of glucose and fat while increasing oxidative stress [23]. In our study, BCAA intake, including leucine, isoleucine, and valine, was lower in individuals with MetS than in those without MetS. However, it was not significantly associated with MetS in the adjusted logistic regression analysis. Leucine and valine intake were also lower in the High-PC group compared to the Low-PC group. Interestingly, BCAA intake did not correlate with serum BCAA and propionylcarnitine concentrations. Therefore, increased serum BCAA and propionylcarnitine concentrations may be linked to metabolic disturbances influenced by genetic factors, insulin resistance, and mitochondrial dysfunction and not to increased intake of BCAA.

Despite the associations observed in this study, it is crucial to recognize that serum propionylcarnitine concentrations are influenced by a complex interplay of genetic, metabolic, and environmental factors. However, there have been limited investigations of the genetic impact on serum propionylcarnitine concentrations and how genetics interact with lifestyle factors to affect these concentrations. The genetic variants associated with serum propionylcarnitine concentrations have been studied with respect to propionic acidemia [24]. In this study, we found that several genetic variants interacted with each other, including *MCTP1*_rs4290997, *KIF7*_rs2350480, *F2*_rs2070850, *PEX3*_rs223231, *TBC1D22A*_rs910543, and *PLA2G4C*_rs7252136. *MCTP1* is a marker for macrophage-1, which may have implications for immunity [25]; *PEX3* is related to oxidative stress [26]; *F2* is linked to diabetic conditions [27]; *PLA2G4C* plays a role in the hydrolysis of membrane phospholipids, specifically phosphatidylcholine, which can affect inflammation and MetS [28]. *TBC1D22A* is involved in cellular processes related to intracellular trafficking and the regulation of cellular metabolism, although research in the context of MetS is limited. These genes in the 6-SNP model appear to be primarily associated with inflammation, which, in turn, can impact metabolic disorders, primarily glucose and lipid metabolism. Other genes among the selected genetic variants are related to similar metabolic processes. *CMKLR1* is linked to nuclear factor kappa-light-chain-enhancer of activated B cells (NF-κB) activation and insulin signaling [29], and *kinesin family member 5B (KIF5B)* is associated with insulin resistance and obesity [30]. *KIF5B* plays a role in growth factors and transforming the growth factor-beta (TGF-β) pathways [31]. Integrating these findings with previous research suggests that the genetic variants selected for their association with high serum propionylcarnitine concentration may be linked to energy metabolism and inflammation, potentially increasing the risk of developing MetS.

The gene *CELSR2*, known for its involvement in cell adhesion and signaling, plays a crucial role in suppressing lipid accumulation and thus mitigating nonalcoholic fatty liver disease (NAFLD), as reported in prior research [32]. Notably, a specific genetic variant, rs12740374, has been linked to atherosclerotic cardiovascular disease [33]. Our current study focused on the *CELSR2_rs629301* located in the 3’UTR, emphasizing its potential role in gene expression regulation. Our findings suggest that this variant interacts with certain food components, altering binding energy and potentially influencing *CELSR2* expression. Moreover, we observed a positive correlation between *CELSR2_rs629301* and serum propionylcarnitine concentrations, revealing a 1.3-fold increase. Interestingly, various flavonoids and their glycones exhibited distinct binding energies with *CELSR2_rs629301*. This information is valuable, as it implies that dietary intake of these compounds may differentially modulate serum propionylcarnitine concentrations in adults carrying the *CELSR2_rs629301* variant. These results have implications for developing personalized nutrition strategies that could effectively intervene in the management of metabolic disorders. 

Our study found that the PRS derived from a combination of six genetic variants interacted with dietary patterns, particularly the diet high in beans, potatoes, and kimchi (the high BPKD). Interestingly, the participants with high BPKD reduced the influence of the PRS on serum propionylcarnitine levels, especially in participants with a medium PRS. However, we did not observe similar interactions between the PRS and other dietary patterns. Smoking also played a significant role in modifying the effect of the PRS on serum propionylcarnitine concentration. It is worth noting that no prior studies have explored how genetic factors interact with lifestyle choices and serum propionylcarnitine concentrations. Hence, our findings are novel. Overall, our study suggests that serum propionylcarnitine concentrations are associated with the metabolism of BCAAs, and a combination of genetic and environmental factors influences these concentrations.

The limitations of our study are as follows: (1) the study employed a cross-sectional design, which limits its ability to establish causality. (2) The participants were Koreans, and, hence, the study results can only be generalized to the East Asian population. (3) Normal food intake was determined using an SQFFQ containing 106 typical Korean dishes, considering the dietary habits over the past year from the interview date. The validity of the SQFFQ was established through comparison with three-day dietary records collected for each of the four seasons. Therefore, it could be underestimated or overestimated. However, this study had some strengths: (1) a relatively large group of participants (*n* = 2580) was included to provide robust statistical power. (2) Both serum BCAA concentrations and BCAA intake were measured to show their relationship with serum propionylcarnitine concentrations and the genetic impact. (3) The study explored the interaction between genetics and dietary patterns and the influence of smoking on serum propionylcarnitine concentrations. This novel approach adds to our understanding of the complex genetic and lifestyle factors affecting serum propionylcarnitine concentrations. Therefore, the present study elucidated the complex interplay between genetic factors, dietary patterns, and smoking in relation to serum propionylcarnitine concentrations. However, despite its cross-sectional nature and the potential for unexplored confounding factors, the results of the present study should be interpreted with caution. 

## 4. Materials and Methods

### 4.1. Participants

In 2001, Korean middle-aged and elderly individuals aged 40–69 years residing in either Ansan (city community) or Ansung (rural community) for a minimum of 6 months participated in the Korean Genome and Epidemiology Study (KoGES), a large population-based study [34]. The KoGES was conducted by the Korea National Institute of Health (KNIH) as part of the Korea Association Resource (KARE) initiative. The study examined demographic, biochemical, and lifestyle factors along with extensive genome-wide genotyping of a large Ansan/Ansung cohort (*n* = 8845). Of the cohort, 2580 participants whose serum metabolite concentrations were determined were included in the present analysis. The KoGES study’s research protocol obtained approval from the institutional review board of the KNIH (KBP-2019-055), and all participants furnished written informed consent. Additionally, the study received approval from the review board of Hoseo University (1041231-150811-HR-034-01).

### 4.2. Demographic Data, Anthropometric and Biochemical Measurements

The demographic and lifestyle-related information of the participants, including age, education, income, medical history, smoking status, coffee and alcohol consumption, and physical activity, were recorded through a health interview. Height, weight, and waist circumference were recorded using a standardized methodology described earlier [19]. The measurements of anthropometric and biochemical parameters were described in previous studies.

### 4.3. History of Lifestyle-Related Factors

The evaluation of coffee and alcohol consumption involved documenting the frequency of consuming one or more servings of coffee or alcohol daily in the previous year before the interview. We multiplied the reported frequency by the volume of coffee or alcohol consumed in a single instance to quantify their intake. Smoking status was assessed and divided into three categories: current, former, and never-smokers. The threshold for distinguishing between current and former smokers was established, with current smokers defined as those smoking more than 100 cigarettes during their lifetime and within the past 6 months. Regular physical activity was determined based on the duration and intensity of exercise. Moderate exercise included activities like doubles tennis, gentle swimming, volleyball, or occupational and recreational endeavors with light objects, performed consistently for over 30 min at least five times per week. Vigorous exercise comprised activities such as fast cycling, brisk swimming, running, climbing, football, basketball, singles tennis, skipping, squash, and occupational or recreational tasks with heavy objects engaged in for more than 20 min at least three times per week.

### 4.4. Assessment of Food and Nutrient Intake by a Semi-Quantitative Food Frequency Questionnaire (SQFFQ)

A specially designed and validated SQFFQ tailored for Korean adults was utilized to assess dietary intake in this study. The SQFFQ aimed to capture the participants’ consumption of diverse food items. A total of 2580 participants completed the SQFFQ, responding to queries about consuming 106 distinct food items. According to the Korean food composition table, the intake of these food items was then converted into 23 nutrients. A validation process for the SQFFQ was conducted by comparing it with 3-day food records collected during the four seasons. This validation process was instrumental in ensuring the reliability of the questionnaire’s outcomes. The average daily nutrient intake from the SQFFQ responses was computed using the computer-aided nutritional analysis software (CAN-Pro) version 3.0. This nutrient database, developed by the Korean Nutrition Society (KNS, Seoul, Korea), facilitated the data analysis of consumed foods.

### 4.5. Determination of Serum Metabolite Concentrations

The participants’ serum metabolites (*n* = 2580) were quantitatively determined through a targeted metabolomics approach utilizing the AbsoluteIDQ^TM^ p180 kit UHPLC column (Biocrates Life Sciences AG, Innsbruck, Austria). The running solvent was acetonitrile and water with 0.2% formic acid. In this process, serum was introduced into the kit for the subsequent extraction of metabolites. Following extraction and centrifugation of the extracts, acylcarnitines were analyzed using flow injection analysis/tandem mass spectrometry (FIA–MS/MS) in the positive ion mode. Amino acids were quantified via liquid chromatography/tandem mass spectrometry (LC–MS/MS) in the positive ion mode. Stable isotope-labeled internal standards (^13^C or ^15^N) were utilized as references to ensure accurate identification and quantification of all metabolites. The measurement of metabolite concentrations in micromolar (µM) units was automated using the MetVal^TM^ software package 2.4 from Biocrates Life Sciences AG (Aliso Viejo, CA, USA). Quality assurance measures included utilizing calibration standards, quality control (QC) samples, and reference standards from normal human pooled serum on each plate. The data quality of each metabolite was verified as described previously [35].

### 4.6. Genotyping and Quality Control

The Center for Genome Science, KNIH, provided the genotype data. Genomic DNA was extracted from whole blood samples, and genotyping was performed using the Affymetrix Genome-Wide Human SNP Array 5.0 (Affymetrix, Santa Clara, CA, USA). The quality and accuracy of the genotyping were assessed using the Bayesian robust linear model with Mahalanobis distance classifier (BRLMM) [36]. Genotypes that did not meet specific criteria were excluded, including those with low genotyping accuracies (<98%), high rates of missing genotype calls (≥4%), excessive heterozygosity (>30%), gender biases, or deviations from the Hardy–Weinberg equilibrium (HWE) (*p* < 0.05) [36]. 

### 4.7. GWAS for Risk of Serum Hyper-Propionylcarnitine Concentrations and Interaction between Genetic Variants by a Generalized Multifactor Dimensionality Reduction (GMDR) Method

Genome-wide association studies (GWASs) were performed on the Ansan/Ansung cohort to investigate the genetic impact on serum hyper-propionylcarnitine concentrations. The analysis was adjusted for age, gender, city residence area, job history in chemical- and dust-related companies, BMI, energy and alcohol intake, physical exercise, and smoking using PLINK (http://pngu.mgh.harvard.edu/~purcell/plink; accessed on 20 March 2023). We evaluated the quality of genetic variants in relation to serum hyper-propionylcarnitine concentrations by validating them using Manhattan and quantile–quantile (QQ) plots. The QQ plot’s lambda value closely approached one, indicating that the genotypes obtained from GWAS were appropriate. We excluded SNPs with high linkage disequilibrium (LD) (D′ > 0.2, r^2^ > 0.001) from further analysis using Haploview 4.2 in PLINK because they contained redundant genetic information [17]. To investigate interactions among these genetic variants, we applied the generalized multifactor dimensionality reduction (GMDR) method, a nonparametric genetic model designed to detect and characterize nonlinear interactions among discrete genetic attributes. Using GMDR analysis to enhance our understanding of the genetic impact, we identified the best model illustrating interactions associated with the risk of serum hyper-propionylcarnitine levels. The criteria for selecting the best model included a significant *p*-value (*p* < 0.05) for the sign test of trained balance accuracy (TRBA) and test balance accuracy (TEBA) along with a cross-validation consistency (CVC) score of 9 or 10 out of 10 [17]. From the GMDR models, we chose the 6-SNP and 7-SNP models to calculate genetic impact. The polygenic risk scores (PRSs) for these models were determined by summing the number of risk alleles of the 6- and 7-SNPs. The calculated PRSs were divided into three categories (0–5, 6–7, and ≥8 for the 6-SNP model and 0–6, 7–8, and ≥9 for the 7-SNP model) as the low-PRS, medium-PRS, and high-PRS groups, respectively. 

### 4.8. Genotype-Tissue Expression (GTEx) of Genetic Mutations

The Genotype-Tissue Expression (GTEx) project leverages the Functional Mapping and Annotation of Genetic Associations (FUMA) web application’s source code. GTEx provided us with normalized gene expression data, quantified as reads per kilobase of transcript per million mapped reads (RPKM), across 53 tissue types. GTEx encompassed a comprehensive catalog of 56,320 genes, from which we refined our analysis by selecting tissues with an average RPKM greater than or equal to one in at least one tissue type.

### 4.9. Molecular Docking of CELSR2 Possessing 3′UTR Mutation with Food Compounds

The sequences of the *Cadherin EGF LAG seven-pass G-type receptor 2* (*CELSR2*) 3′ UTR, including rs629301, were received from the ensemble genome browser website, and its RNA structure was generated using https://rnacomposer.cs.put.poznan.pl/. The wild and mutated protein data bank (PDB) formats were converted into PDB, partial charge (Q), and atom type (T) (PDBQT) files using AutoDock Tools 1.5.6 (Molecular Graphics Laboratory, Scripps Research Institute, Jupiter, FL, USA) [37]. The PDBQT format of the *CELSR2* 3′ UTR, including rs629301, was simulated with about 20,000 food components (*n* = 20,000). The food components were selected when the binding energy between the *CELSR2* 3′ UTR, including rs629301, and the food components was less than −10.0 kcal/mol [38]. The lower the binding free energy, the tighter the binding and the greater the affinity.

### 4.10. Statistical Analysis

We employed SAS (version 9.3; SAS Institute, Cary, NC, USA) for statistical analysis of the data. To assess the categorical variables among participants, including gender, education levels, smoking status, and various parameters, we examined the frequency distributions based on the tertiles of the 6-SNP PRS categories: low, medium, and high groups (≤5, 6–7, and ≥8). A chi-squared test was used to assess the frequency distributions of the categorical variables. In addition, we conducted one-way analysis of variance (ANOVA) to compare the PRS categories across continuous variables like age, energy intake, and others.

The association between anthropometric, metabolic, and genetic parameters with the risk of serum hyper-propionylcarnitine concentrations was examined using adjusted logistic regression analysis. Two different models, referred to as model 1 and model 2, with different covariates were employed. The first model adjusted for the residence area, gender, age, and BMI. In the second model, we expanded the adjustments from model 1 to include smoking and drinking status, total physical activity, and daily energy intake as covariates. We calculated odds ratios (ORs) along with 95% confidence intervals (CIs) while using a low PRS as the reference.

The potential interaction between the PRS and lifestyle factors that influence the risk of serum hyper-propionylcarnitine concentrations was evaluated with a multivariate general linear model (GLM) analysis with the main effects of PRS and lifestyle and covariates. In cases where a significant interaction was observed in the multivariate GLM, logistic regression analysis was performed in two groups based on cutoff values assigned to each lifestyle parameter. The cutoff values were determined by the ≤25th percentile for the low level, while the remaining values were classified as high. This classification was established with the assumption that a lower level of each parameter had a greater probability of interacting with the PRS. To determine specific cutoff values for each parameter, we referred to either the recommended intake or the 33rd percentile of intake as outlined in the Korean dietary reference intake [39]. Using these classification criteria, participants were subsequently divided into high and low groups based on their lifestyle parameters. We considered a *p*-value of ≤0.05 as the threshold for statistical significance. 

## 5. Conclusions

Our study revealed the association between elevated serum propionylcarnitine levels and BCAA metabolism, underscoring its potential as an indicator for MetS and cardiovascular risk. We also emphasize the intricate interplay involving genetic factors, dietary patterns such as a high BPKD, and smoking in regulating serum propionylcarnitine concentrations. For individuals with medium and high PRS related to serum propionylcarnitine concentrations, adopting a high BPKD and avoiding smoking may be advisable to mitigate the genetic impact and reduce the risk of elevated serum propionylcarnitine levels. However, we acknowledge that further research is essential to fully elucidate the underlying mechanisms linking propionylcarnitine metabolism and MetS. Specifically, we need to explore the mechanism of genetic factors governing its regulation and assess the differences between individuals with MetS and those without. A comprehensive understanding of these relationships holds the potential to enhance risk assessment and intervention strategies, particularly for individuals at risk of MetS and its associated health complications, including cardiovascular diseases.

## Figures and Tables

**Table 1:** Demographic characteristics regarding metabolic syndrome (MetS) and its components.

	Non-MetS (*n* = 2001)	MetS (*n* = 579)	Adjusted OR and 95% CI
Age (years) ^1^	52.4 ± 0.18	54.0 ± 0.31 ***	1.495 (1.088–2.056)
Gender (male, %)	910 (46.9)	313 (48.9)	1.034 (0.728–1.468)
Education (number, %)			
Middle school High school >High school	1108 (57.5) 566 (29.4) 252 (13.1)	387 (61.1) 179 (28.1) 68 (10.7)	1 0.933 (0.701–1.243) 0.804 (0.546–1.184)
Residence area (number, %)			
Rural City	1074 (55.4) 866 (44.6)	356 (55.6) 284 (44.4)	1 1.316 (0.979–1.769)
Physical activity (Yes, Number, %) ^2^	1051 (56.2)	350 (56.7)	0.982 (0.779–1.237)
Alcohol (g/day) ^3^	9.41 ± 0.45	11.2 ± 0.79 *	1.377 (1.014–1.870)
Nonsmoker (Number, %) Former Current	1143 (59.6) 284 (14.8) 491 (25.6)	363 (57.4) 104 (16.4) 166 (26.2)	1 0.968 (0.652–1.438) 0.963 (0.670–1.384)

The values represent means ± standard errors or number of the subjects (percentage of each group). Adjusted odds ratio (OR) and 95% confidence intervals (CIs) with the covariates adjusting for age, gender, body mass index, physical activity, education, smoking, and intake of alcohol, dietary fiber, and energy. The cutoff points of the reference were as follows: ^1^ <55 years old for age, rural area, ^2^ <moderate intensity exercise, ^3^ <20 g/day alcohol intake. Asterisks Significantly different from the non-MetS (normal-control) group at * *p* < 0.05 and *** *p* < 0.001.

**Table 2:** Serum carnitine, acylcarnitine, and amino acid concentrations between metabolic syndrome (MetS) and non-MetS.

	Non-MetS (*n* = 2001)	MetS (*n* = 579)	Adjusted OR and 95% CI
Carnitine (µM)	50.4 ± 0.24	50.7 ± 0.46	1.074 (0.852–1.353)
Acetylcarnitine (µM)	7.06 ± 0.06	6.81 ± 0.11 *	0.774 (0.612–0.979)
Propionlycarnitine (µM)	0.455 ± 0.004	0.479 ± 0.007 **	1.454 (1.163–1.817)
Butanoylcarnitine (µM)	0.201 ± 0.002	0.209 ± 0.004	1.138 (0.912–1.421)
Varelyoylcarnitine (µM)	0.149 ± 0.002	0.155 ± 0.003	1.262 (1.003–1.590)
Valine (µM)	217 ± 0.93	231 ± 1.75 ***	1.908 (1.530–2.378)
Isoleucine (µM)	81.8 ± 0.43	87.8 ± 0.82 ***	1.983 (1.580–2.489)
Leucine (µM)	173 ± 0.80	182 ± 1.52 ***	1.498 (1.194–1.880)
BCAA (µM)	595 ± 2.45	622 ± 4.63 ***	1.531 (1.224–1.916)
Methionine (µM)	24.2 ± 0.15	25.1 ± 0.28 **	1.139 (0.903–1.437)
Threonine (µM)	142 ± 0.76	139 ± 1.44	0.979 (0.778–1.232)
Tyrosine (µM)	71.0 ± 0.34	74.6 ± 0.64 ***	1.597 (1.283–1.988)
Lysine (µM)	205 ± 1.02	208 ± 1.94	1.046 (0.833–1.313)
Alanine (µM)	496 ± 2.69	538 ± 5.09 ***	1.972 (1.591–2.444)
Tryptophan (µM)	62.4 ± 0.30	63.5 ± 0.56	1.220 (0.970–1.536)
Dietary valine (g/day)	3.43 ± 0.18	3.33 ± 0.34 *	0.903 (0.627–1.300)
Dietary isoleucine (g/day)	2.70 ± 0.17	2.61 ± 0.33 *	0.814 (0.582–1.138)
Dietary leucine (g/day)	4.23 ± 0.028	4.11 ± 0.052 *	0.873 (0.607–1.254)
Dietary BCAA (g/day)	10.4 ± 0.06	10.0 ± 0.118 *	0.849 (0.597–1.209)
Dietary methionine (g/day)	9.57 ± 0.011	9.22 ± 0.021	0.992 (0.728–1.352)
Dietary tyrosine (g/day)	1.46 ± 0.013	1.42 ± 0.026	1.011 (0.735–1.391)

The values represent means ± standard errors or number of the subjects (percentage of each group). Adjusted odds ratio (OR) and 95% confidence intervals (CIs) with adjusting for the covariates such as age, gender, BMI, residence area, physical activity, education, smoking, and intake of alcohol, dietary fiber, and energy. The cutoff point for the reference in all parameters for the logistic regression was the 33rd percentile. Asterisks Significantly different from the non-MetS (normal-control) group at * *p* < 0.05, ** *p* < 0.01, *** *p* < 0.001. BCAA: branched-chain amino acids.

**Table 3:** Metabolic parameters related to metabolic syndrome (MetS) according to serum propionlycarnitine (PC) concentrations.

	Low-PC (*n* = 1918)	High-PC (*n* = 633)	Adjusted ORs and 95% CI
MetS (number, %)	406 (21.0)	173 (26.9) **	1.389 (1.107–1.742)
Body mass index (kg/m^2^) ^1^	24.6 ± 0.08	25.1 ± 0.15 *	1.250 (1.025–1.525)
Waist circumferences (cm) ^2^	82.8 ± 0.20	84.4 ± 0.36 ***	1.417 (1.057–1.899)
Body fat (%) ^3^	26.7 ± 0.14	27.5 ± 0.24 **	1.298 (1.055–1.598)
Lean body mass (%) ^4^	69.5 ± 0.12	66.6 ± 0.24 **	0.581 (0.382–0.883)
Serum glucose at 0 min (mg/dL) ^5^	87.8 ± 0.45	87.3 ± 0.78	1.180 (0.837–1.663)
Serum glucose at 60 min (mg/dL) ^6^	154 ± 1.23	159 ± 2.22	1.258 (1.039–1.523)
Serum glucose at 120 min (mg/dL) ^7^	125 ± 1.23	140 ± 2.51 ***	1.041 (0.847–1.279)
HbA1c (%) ^8^	5.74 ± 0.02	5.84 ± 0.03 **	1.314 (0.950–1.817)
Serum insulin at 0 min (mU/L) ^9^	7.81 ± 0.11	8.01 ± 0.20	1.209 (0.968–1.509)
Serum insulin at 60 (mU/L) ^10^	31.8 ± 0.78	34.5 ± 1.39	1.076 (0.867–1.336)
Serum insulin at 120 (mU/L) ^11^	27.7 ± 0.69	31.3 ± 1.24 *	1.277 (1.029–1.586)
HOMA-IR ^12^	1.69 ± 0.03	1.76 ± 0.05 *	1.345 (1.112–1.626)
HOMA-B ^13^	156.2 ± 3.56	144.9 ± 6.40	0.907 (0.738–1.113)
Serum total cholesterol (mg/dL) ^14^	194 ± 0.82	195 ± 1.46	0.956 (0.732–1.249)
Serum HDL-C (mg/dL) ^15^	45.2 ± 0.23	43.3 ± 0.41 ***	1.387 (1.131–1.701)
Serum LDL-C (mg/dL) ^16^	116.1 ± 0.77	115.9 ± 1.37	0.953 (0.771–1.177)
Serum TG (mg/dL) ^17^	161.3 ± 2.55	179.5 ± 4.55 **	1.586 (1.309–1.922)
SBP (mmHg) ^18^	117.8 ± 0.39	120.8 ± 0.69 ***	1.336 (1.065–1.675)
DBP (mmHg) ^19^	75.4 ± 0.25	78.0 ± 0.45	1.870 (1.436–2.435)
CHD (number, %)	30 (1.55)	21 (3.27) **	1.906 (1.025–3.544)

The values represent means ± standard errors or number of the subjects (percentage of each group). Adjusted odds ratio (ORs) and 95% confidence intervals (CIs) with the adjusting for the covariates such as age, gender, BMI, residence area, physical activity, education, smoking, and intake of alcohol, dietary fiber, and energy. The cutoff points of the reference were as follows: ^1^ <25 kg/m^2^; ^2^ <90 cm for men and 85 cm for women; ^3^ <25% for men and 30% for women; ^4^ <71%; ^5^ <110 mg/dL plus antidiabetic medication; ^6^ <160 mg/dL; ^7^ <140 mg/dL; ^8^ <6.5%; ^9^ <9.8 mU/L; ^10^ <43.0 mU/L; ^11^ < 39 mU/L; ^12^ <1.45; ^13^ <150; ^14^ <230 mg/dL; ^15^< 40 mg/dL for men and <50 mg/dL for women plus dyslipidemia medication; ^16^ <130 mg/dL; ^17^ <150 mg/dL; ^18^ <140 mmHg; ^19^ <90 mmHg plus hypertension medication. Asterisks Significantly different from the non-MetS (normal-control) group at * *p* < 0.05, ** *p* < 0.01, *** *p* < 0.001. SBP: systolic blood pressure; DBP: diastolic blood pressure; HOMA-IR, homeostatic model assessment for insulin resistance; HOMA-B, HOMA for insulin secretion; HDL-C: high-density lipoprotein cholesterol; LDL-C: low-density lipoprotein cholesterol; TG: triglycerides; CHD: cardiovascular disease; HbA1c: glycosylated hemoglobin.

**Table 4:** The characteristics of the 10 genetic variants related to serum hyper-propionylcarnitine concentrations used for the generalized multifactor dimensionality reduction analysis.

Chr ^1^	SNP ^2^	Position	Mi ^3^	Ma ^4^	OR ^5^	SE	^6^ *p*-Value Adjusted	Gene Names	Functional Consequence	^7^ MAF	^8^ *p*-Value for HWE
1	rs629301	109818306	G	T	1.64	0.127	9.17 × 10^−6^	*CELSR2*	3′ UTR	0.059	0.295
5	rs4290997	94430813	T	C	1.34	0.07	2.84 × 10^−5^	*MCTP1*	Intron	0.402	0.74
5	rs153197	132727692	T	C	0.594	0.126	3.65 × 10^−5^	*FSTL4*	Intron	0.108	0.408
6	rs223231	143781637	C	G	1.382	0.082	8.16 × 10^−6^	*PEX3*	Intron	0.201	0.716
11	rs2070850	46741495	C	T	1.343	0.07	2.51 × 10^−5^	*F2*	Intron	0.403	0.507
12	rs7315943	108709475	T	G	3.102	0.272	3.17 × 10^−5^	*CMKLR1*	Intron	0.015	0.725
15	rs2350480	90203253	A	C	1.334	0.072	4.67 × 10^−5^	*KIF7*	Intron	0.326	0.561
16	rs9933938	83699603	A	T	1.66	0.11	3.69 × 10^−6^	*CDH13*	NMD transcript	0.094	0.851
19	rs7252136	48604234	C	T	0.666	0.102	7.25 × 10^−6^	*PLA2G4C*	NMD transcript	0.149	0.802
22	rs910543	47556391	G	A	0.718	0.085	9.46 × 10^−5^	*TBC1D22A*	NMD transcript	0.249	0.363

^1^ Chromosome; ^2^ single-nucleotide polymorphism; ^3^ minor allele; ^4^ major allele; ^5^ odds ratio; standard errors; ^6^ *p*-value for OR after adjusting for age, gender, BMI, residence area, physical activity, education, smoking, and intake of alcohol, dietary fiber, and energy; ^7^ minor allele frequency; ^8^ Hardy–Weinberg equilibrium. *CELSR2; Cadherin EGF LAG seven-pass G-type Receptor 2, MCTP1; multiple C2 and transmembrane domain containing 1, FSTL4; Follistatin-like 4, PEX3; Peroxisomal Biogenesis Factor 3, F2; Coagulation Factor II, (Thrombin), CMKLR1; Chemerin-like Receptor 1, KIF7; Kinesin Family Member 7, CDH13; H-Cadherin, PLA2G4C; Phospholipase A2 Group IV C, TBC1D22A; TBC1 Domain Family Member 22A.*

**Table 5:** Adjusted odds ratios (ORs) for the risk of serum hyper-propionylcarnitine concentrations by polygenic risk scores (PRSs) of the best model after covariate adjustments according to low- and high-lifestyle factors.

	Low PRS	Medium PRS	High PRS	*p*-Value for Interaction
Low-serum BCAA ^1^ High-serum BCAA	1 1	1.294 (0.544–3.081) 2.193 (1.668–2.883)	2.928 (1.070–8.015) 3.933 (2.789–5.545)	0.0452
Low-KBD ^2^ High-KBD	1 1	2.043 (1.583–2.636) 2.021 (1.178–3.467)	3.711 (2.699–5.102) 3.477 (1.734–6.969)	0.7085
Low-NBFD ^2^ High-NBFD	1 1	1.966 (1.576–2.453) 2.119 (1.234–3.640)	3.221 (2.432–4.266) 4.164 (2.133–8.130)	0.9681
Low-BPKD ^2^ High-BPKD	1 1	2.076 (1.604–2.686) 1.176 (0.704–1.966)	3.762 (2.729–5.186) 3.956 (2.156–7.259)	0.0044
Low-RMD ^2^ High-RMD	1 1	1.977 (1.585–2.466) 1.939 (1.199–3.135)	3.229 (2.439–4.276) 2.680 (1.458–4.923)	0.6944
Nonsmoker Smoker	1 1	1.749 (1.230–2.488) 2.645 (1.803–3.881)	3.416 (2.223–5.247) 4.677 (2.872–7.615)	0.0686
Low-alcohol ^3^ High-alcohol	1 1	1.857 (1.405–2.456) 2.532 (1.354–4.733)	3.173 (2.233–4.510) 6.494 (2.977–14.16)	0.2214
Low-exercise ^4^ High-exercise	1 1	1.839 (1.368–2.471) 2.555 (1.560–4.183)	3.418 (2.365–4.940) 5.899 (3.170–10.98)	0.3791

Values represent odd ratios and 95% confidence intervals. PRS with 7-SNPs were divided into three categories (1–5, 6–7, and ≥8) by tertiles as the low, medium, and high groups of the best model of GMDR. The cutoff point was as follows: ^1^ <500 µM serum branched-chain amino acids (BCAAs), ^2^ <33rd percentiles; ^3^ <20 g/day alcohol intake, and ^4^ <150 min/week moderate intensity exercise. Multiple logistic regression models include the corresponding main effects, interaction terms of PRS and main effects (lifestyle factors), and potential confounders such as age, gender, BMI, residence area, physical activity, education, smoking, and intake of alcohol, dietary fiber, and energy. The reference was the low PRS. BCAA: branched-chain amino acids; KBD: Korean balanced diet; NBFD: a diet high in noodles, bread, and fast food; BPKD: a diet rich in beans, potato, and kimchi; RMD: a rice-main diet.

## Data Availability

The raw data involved in this study will be available by the authors to any qualified researcher.

## Supplementary Materials

The supporting information can be downloaded at: https://www.mdpi.com/article/10.3390/ijms242115810/s1.
