# Supplementary material for: Genetic and Lifestyle-Related Factors Influencing Serum Hyper-Propionylcarnitine Concentrations and Their Association with Metabolic Syndrome and Cardiovascular Disease Risk
Source: Int J Mol Sci. 2023 Oct 31;24(21):15810. doi: 10.3390/ijms242115810 (PMC10647558; doi:10.3390/ijms242115810)

Table S1. Daily nutrient intake and its association with serum propionylcarnitine (PC) concentrations

	Low-PC (n=1918)	High-PC (n=633)	Adjusted ORs and 95% CI
Energy intake (EER %)[1]	101±0.91	104±1.58	1.085 (0.877-1.342)
CHO (En%)[2]	70.7±0.17	70.4±0.30	1.148 (0.892-1.477)
Fat (En%)[3]	14,6±0.13	14.6±0.23	1.142 (0.927-1.405)
Protein (En%)[4]	13.6±0.06	13.7±0.10	1.038 (0.848-1.271)
Valine (g/day)[5]	3.42±0.18	3.35±0.32*	0.744 (0.518-1.068)
Isoleucine (g/day)[6]	2.70±0.018	2.61±0.03*	0.822 (0.599-1.127)
Leucine (g/day)[7]	4.23±0.28	4.12±0.49	0.779 (0.551-1.103)
BCAA (g/day) [8]	10.3±0.06	10.1±0.11	0.836 (0.589-1.187)
Fiber (g/day) [9]	15.4±0.25	15.5±0.43	0.891 (0.702-1.129)
V-C (mg/day)[10]	118±1.80	120±3.12	1.127 (0.885-1.434)
Ca (mg/day) [11]	466±5.0	476±8.67	1.042 (0.863-1.260)
Na (mg/day) [12]	3112±34.1	3160±59.1	1.137 (0.900-1.436)
V-D (ug/day) [13]	5.90±0.14	6.10±0.24	1.043 (0.803-1.356)
Coffee (cup/ week) [14]	3.7±0.06	3.5±0.09*	0.803 (0.656-0.983)
Tea (ml/day)[15]	34.7±0.66	34.8±1.15	0.836 (0.675-1.035)
Caffeine (mg/day)[16]	16.6±0.16	15.6±0.29**	0.792 (0.631-0.994)
KBD (number, %)[17]	1397 (72.0)	460 (71.9)	1.116 (0.889-1.401)
NBFD (number, %)[17]	1382 (71.2)	468 (73.1)	0.937 (0.751-1.170)
BPKD (number, %)[17]	1380 (71.1)	471(73.6)	0.972 (0.782 1.208)
RMD (number, %)[17]	1442 (74.3)	493 (77.0)	0.957 (0.759 1.206)

The values represent means ± standard errors or number of the subjects (percentage of each group). Adjusted odds ratio (ORs) and 95 % confidence intervals (CI) with the covariates of adjusting for age, gender, BMI, residence area, physical activity, education, smoking, and intake of alcohol, dietary fiber and energy. The cutoff points of the reference were as follows: [1]estimated energy requirement (EER); [2] 65 energy percent (En%); [3]15 En%; [4] 15 en%; [5] 2.5 g/d; [6] 1.9 g/d; [7] 2.9 g/d; [8] 7.2 g/d; [9] 8.9 g/d; [10] 100 mg/d; [11] 500 mg/dL; [12] 2.5 g/d; 13 10 ug/d; [14] 3 cup/wk; [15] 1 cup/d ; [16] 10 mg/d; [17] 33th percentile. *Significantly different from the Low-PC group at P<0.05 and ** P<0.01. EER: Energy efficiency ratio; CHO: Carbohydrates; BCAA: Branched chain amino acids; V-C: Vitamin C; V-D: Vitamin D; Ca: Calcium; Na: Sodium; KBD: Korean balanced diet; NBFD: a diet high in noodles, bread, and fast food; BPKD: a diet rich in beans, potato, and kimchi RMD : Rice main diet .

Table S2. Binding energy between wild and mutated type *CELSR2*_rs629301 and food components

Food components	Wild type (T)	Mutated type (G)
(S)-Salsolinol	-10.3	-11.4
6-Geranylnaringenin	-10.4	-10.4
Apigenin 6-C-galactoside 8-C-arabinoside	-10.2	-10.4
Apigenin 7-O-diglucuronide	-10.4	-10.4
Apigenin 7-O-glucuronide	-10.1	-10.4
Chrysoeriol 7-O-(6"-malonyl-apiosyl-glucoside)	-10.2	-10.4
Ellagic acid acetyl-xyloside	-10.6	-10.2
Ellagic acid arabinoside	-10.1	-10.2
Gallic acid 3-O-gallate	-10.4	-10.1
N-gamma-L-Glutamyl-L-valine	-10.1	-10.1
gamma-L-Glutamyl-S-(2-carboxy-1-propyl)cysteinylglycine	-10.4	-10.1
Geranyl 3-methylbutanoate	-10.1	-10.1
Jaceosidin	-10.1	-11.3
Kaempferol 3-O-(2"-rhamnosyl-6"-acetyl-galactoside) 7-O-rhamnoside	-11	-10.6
Kaempferol 3-O-(6"-malonyl-glucoside)	-10.3	-10.6
Kaempferol 3-O-(6"-acetyl-galactoside) 7-O-rhamnoside	-10.1	-10.6
Kaempferol 3-O-acetyl-glucoside	-10.2	-10.6
Kaempferol 3-O-glucosyl-rhamnosyl-galactoside	-10.1	-10.6
Kaempferol 3-O-glucosyl-rhamnosyl-glucoside	-10.2	-10.6
Procyanidin trimer EEC	-10.5	-10.6
Prodelphinidin trimer GC-C-C	-10.1	-10.6
Quercetin 3-O-acetyl-rhamnoside	-10.2	-10.3
Quercetin 3-O-rhamnosyl-(1->2)-rhamnosyl-(1->6)-glucoside	-10.5	-10.3
Quercetin 3-O-xylosyl-glucuronide	-10.1	-10.3
Quercetin 3-O-xylosyl-rutinoside	-10.5	-10.3
Licoricesaponin G2	-8.9	-10.1
Quercetin 3-(2"-acetylrutinoside)	-8.9	-10.2
GDP-L-fucose	-8.9	-10.2
Pteroyltriglutamic acid	-8.9	-10.2
Methylcobalamin	-8.3	-10.2
Goyaglycoside g	-10.0	-8.4
Crosatoside A	-10.1	-9.2

Table S3. The characteristics of the ten genetic variants of genes in the risk of serum hyperpropylcarnitine concentrations used for the generalized multifactor dimensionality reduction analysis

	Model 1				Model 2			
Genetic variants	TRBA	TEBA	P value	CVC	TRBA	TEBA	P value	CVC
*MCTP1*_rs4290997	0.5234	0.4804	0.989	4	0.5247	0.4977	0.623	7
*KIF7*_rs2350480 model 1	0.5389	0.5004	0.623	4	0.5387	0.501	0.623	5
*F2*_rs2070850 model 2	0.5559	0.5064	0.377	7	0.5557	0.5057	0.377	7
*PLA2G4C*_rs7252136 model 3	0.5849	0.5208	0.055	7	0.5851	0.5263	0.0107	6
*PEX3*_rs223231, *TBC1D22A*_rs910543 model 3	0.6209	0.5098	0.623	6	0.6201	0.5134	0.1719	6
*PLA2G4C*_rs7252136 model 5	0.6649	0.5541	0.0107	10	0.6623	0.546	0.001	10
*FSTL4*_rs153197 model 6	0.7082	0.5445	0.001	10	0.706	0.5376	0.001	10
*CDH13*_rs9933938 model 7	0.7437	0.5323	0.0107	10	0.7422	0.5305	0.001	10
*CELSR2*_rs629301 model 8	0.7735	0.5284	0.0107	10	0.7712	0.5302	0.0107	10
*CMKLR1*_rs7315943 model 9	0.7791	0.523	0.0547	10	0.7767	0.5236	0.0107	10

Model 1: Adjusted for body mass index, residence area, sex, age

Model 2: Adjusted for body mass index, residence area, sex, age, alcohol, physical activity, smoke, physical activity, energy intake

Supplementary figures

Figure S1. The distribution of genetic variants related to serum propionylcarnitine concentrations

A. Manhattan plot of genetic variants related to serum propionylcarnitine concentrations

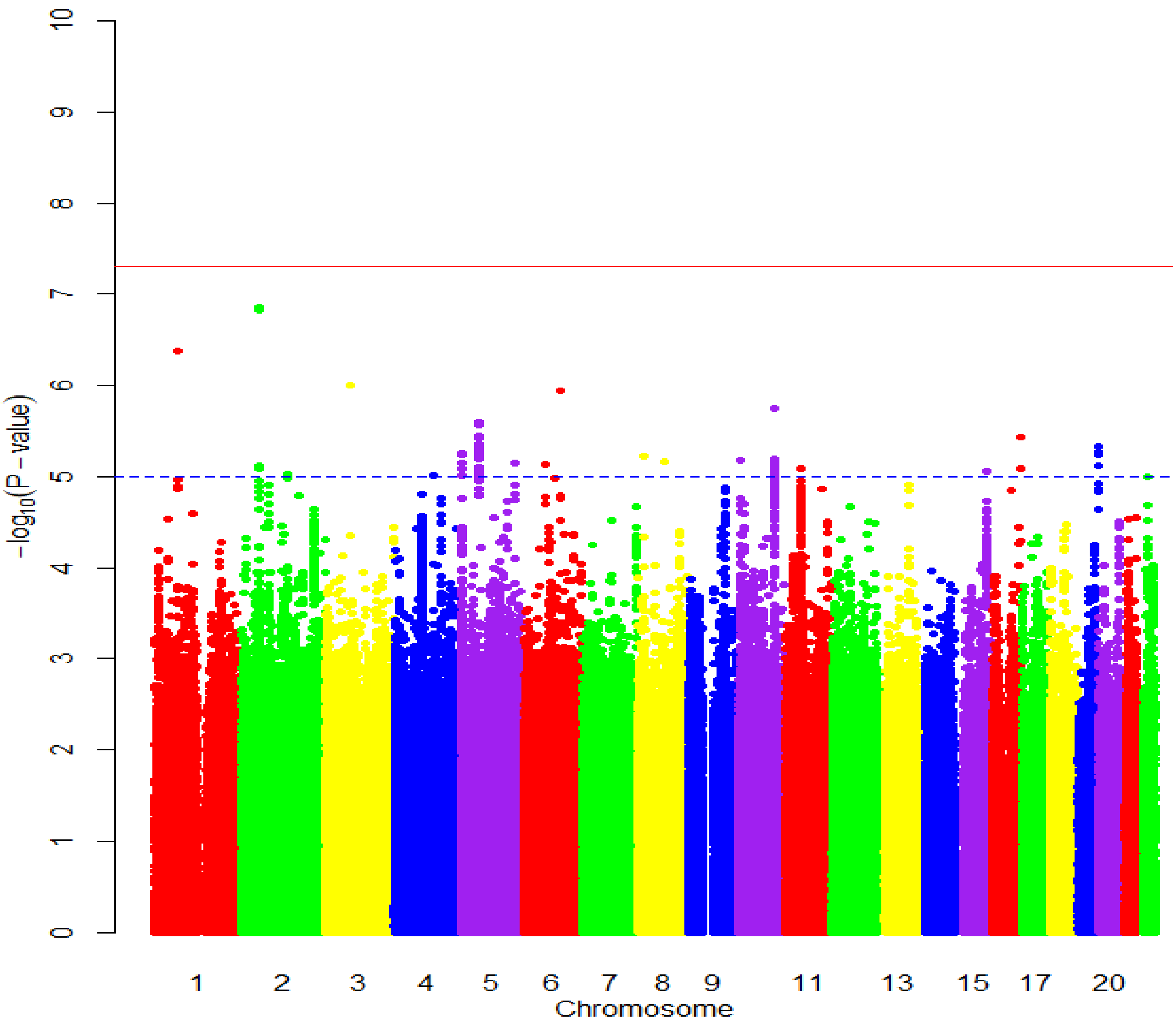

B. Q-Q plot of genetic variants related to serum propionylcarnitine concentrations

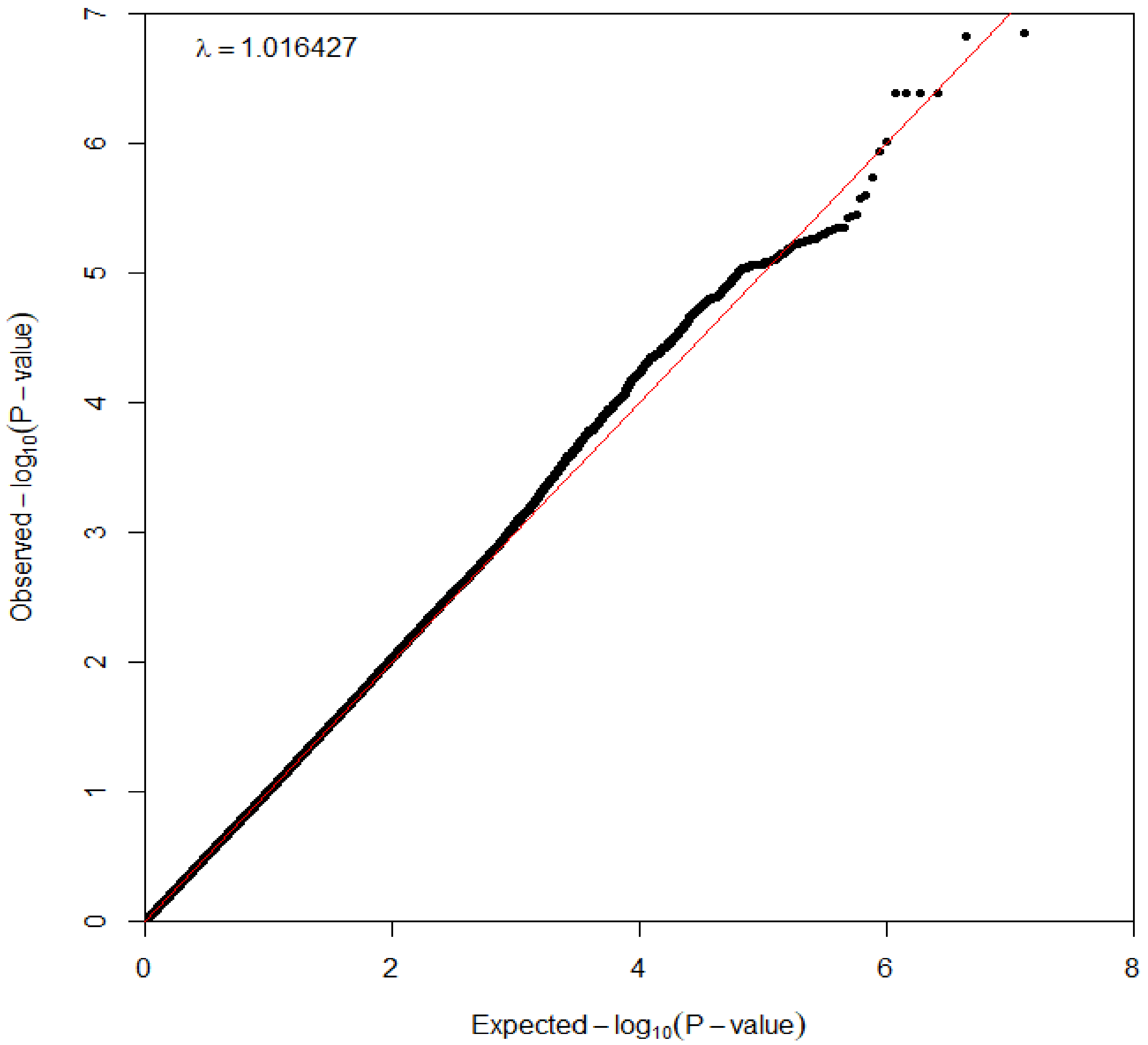

Figure S2. Molecular docking of goyaglycoside g on wild type (WT) and mutated types (MT) of *CELSR2_rs629301 3'UTR*

A. Intermolecular forces between goyaglycoside g on wild type (WT) *CELSR2_rs629301 3'UTR* without the RNA

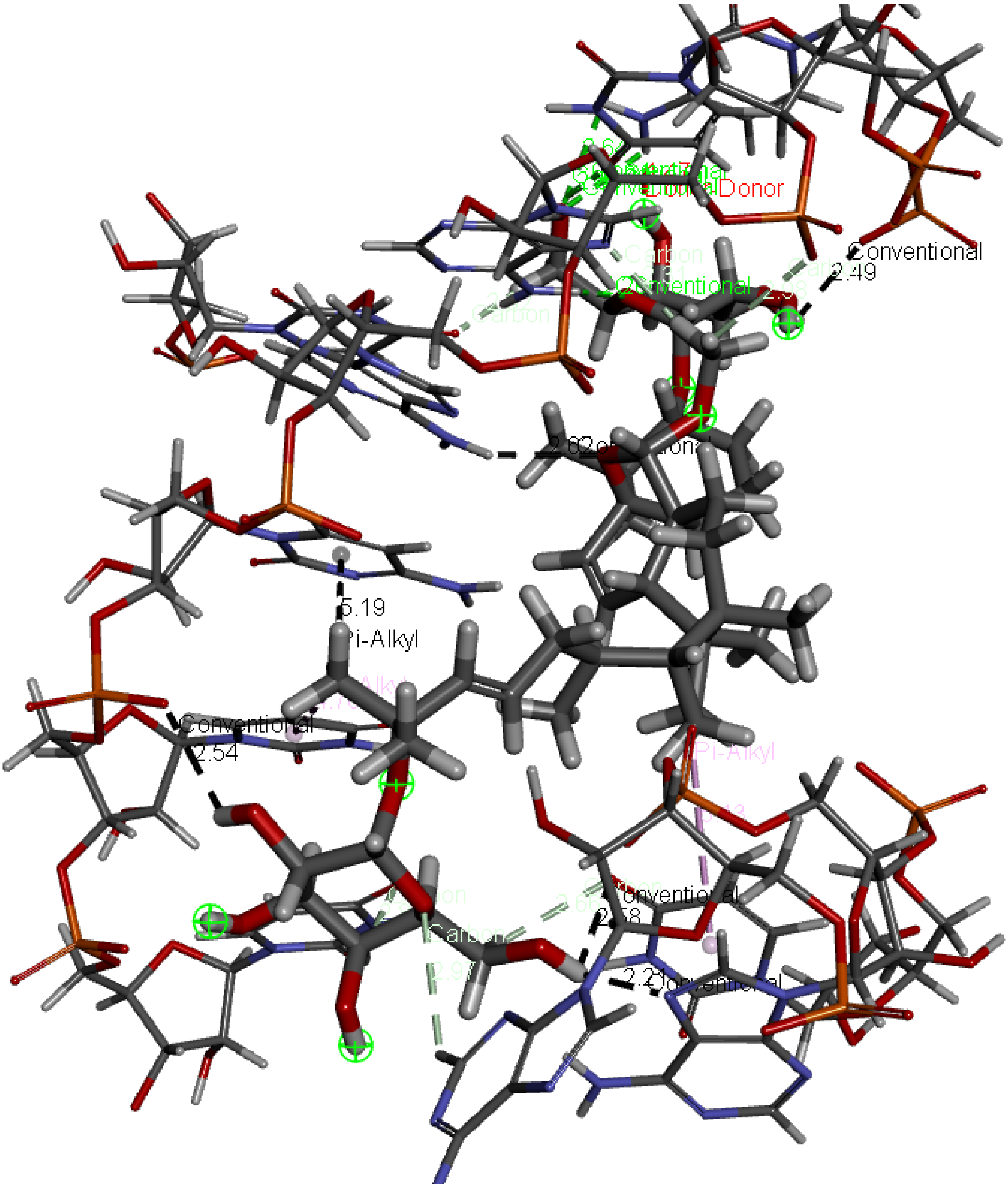

B. Interpolated charge between goyaglycoside g on wild type (WT) *CELSR2_rs629301 3'UTR* without the RNA

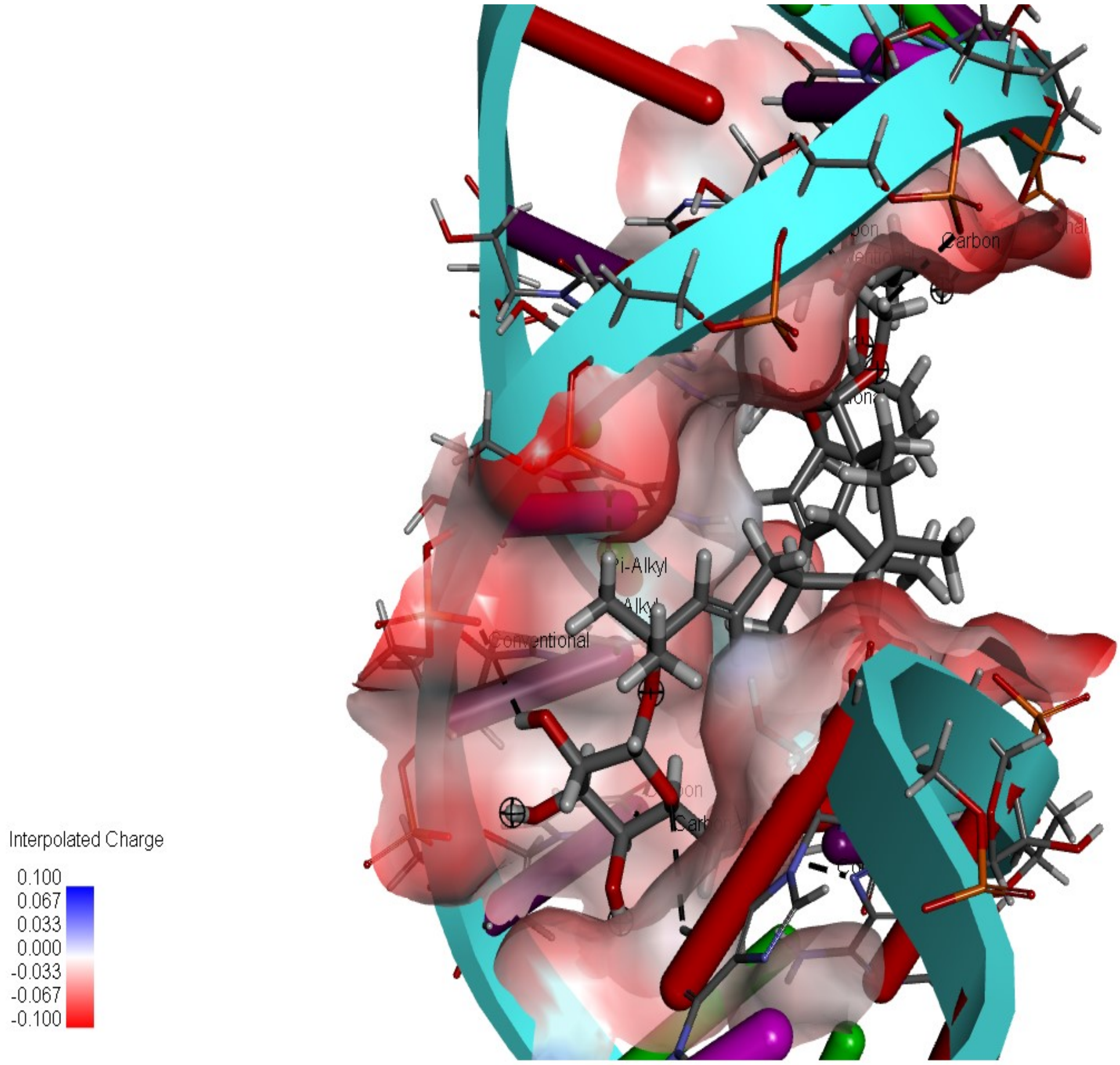

C. Intermolecular forces between goyaglycoside g on mutated type (MT) *CELSR2_rs629301 3’UTR* without the RNA

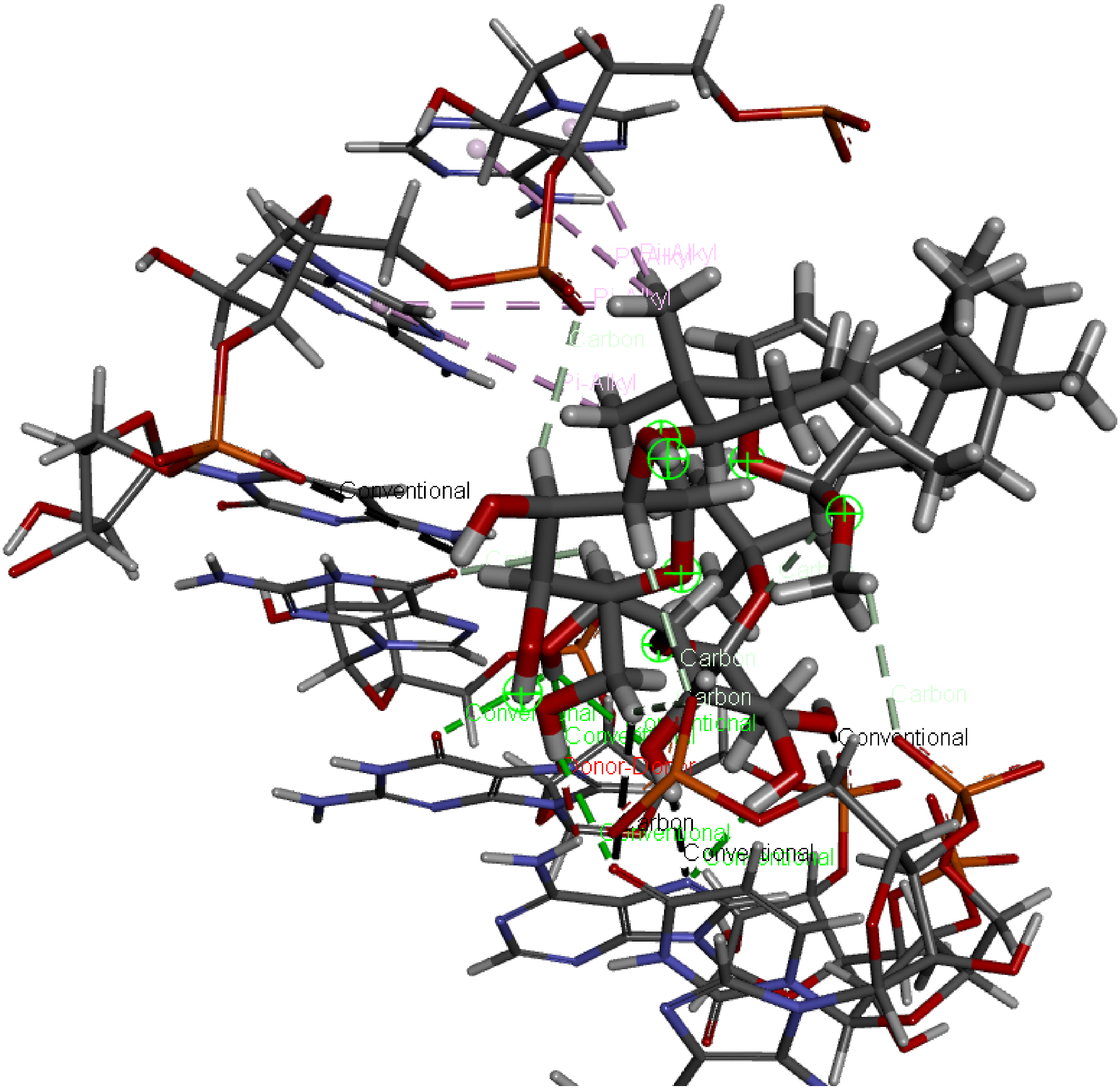

D. Interpolated charge between goyaglycoside g on mutated type (WT) *CELSR2_rs629301 3’UTR* without the RNA

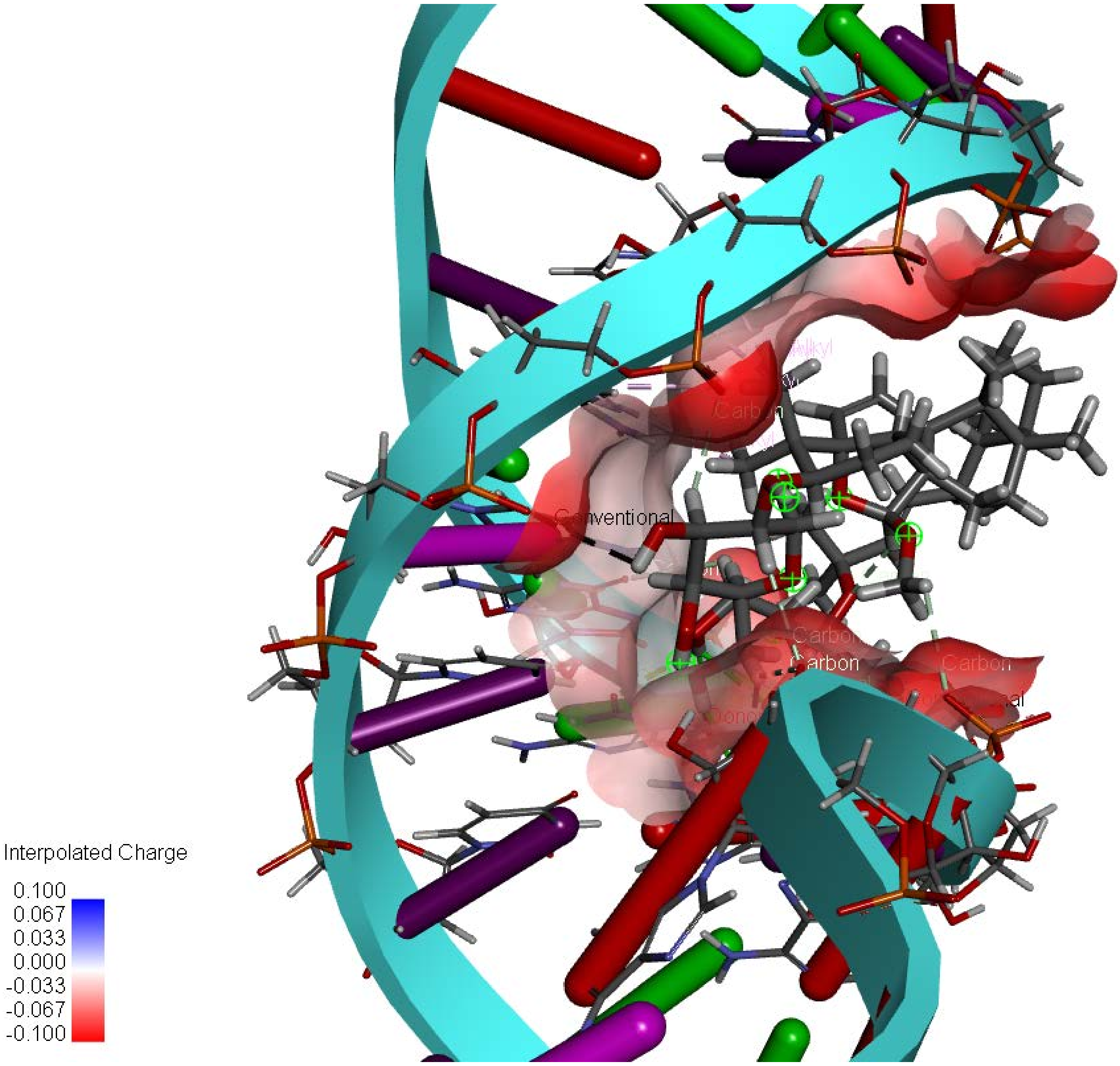

Supplement: Supplementary file 1 [file ijms-24-15810-s001.zip › ijms-2661252-supplementary.pdf]
